# Patients' satisfaction with emergency care services in a University Teaching Hospital in South-West, Nigeria

**DOI:** 10.1016/j.afjem.2021.03.015

**Published:** 2021-04-27

**Authors:** Olabisi Olamide Deji-Dada, Samuel Ayokunle Dada, Johnson Dare Ogunlusi, Olusoji Abidemi Solomon

**Affiliations:** a Department of Family Medicine, Ekiti State University Teaching Hospital, Ado-Ekiti, Nigeria; b Department of Medicine, Ekiti State University Teaching Hospital, Ado-Ekiti, Nigeria; c Accident and Emergency Unit, Ekiti State University Teaching Hospital, Ado-Ekiti, Nigeria

**Keywords:** Patient satisfaction, Emergency care, Healthcare, Emergency Centre

## Abstract

**Background:**

Patient satisfaction is a measure of the extent to which patients are contented with the health care they received from their health care provider.

**Objective:**

The goal of this study was to measure the satisfaction of patients admitted to the Emergency Centre and to determine the factors affecting the satisfaction.

**Method:**

A cross-sectional study was conducted over four months among patients admitted into the Emergency Centre of the hospital. Systematic sampling method was used by trained personnel who collected the data from the participants using a pre-tested structured questionnaire.

**Result:**

Out of 199 patients that participated, 51.3% rated the reception at the Emergency Centre as very good while the speed of pain control was rated as excellent by only 9.0% of the participants. The time to surgical intervention was rated very good and excellent by 57.3% and 9.5% respectively. Comparable value was obtained by both nurses and doctors on the overall attitude across the 5 scoring domains. Overall, 90.5% of participants were satisfied with the services and experiences at the Emergency Centre of the hospital, however, suggested areas of improvement include employment of more staff by 51.8%, provision of more equipment by 41.2%, and 27.6% requested for availability of more facilities.

**Conclusion:**

A high percentage of the patients were satisfied with the overall service in our Emergency Centre while some other areas require improvement.

## African relevance

• Delivering healthcare entails not only getting the patient well but also ensuring that patient's overall experience is satisfactory.
• It is noteworthy that healthcare providers should prioritise key interventions which improve patient satisfaction such as excellent interpersonal and attitudinal skills, effective communication with clients and short waiting time among other factors.
• This report is relevant for physicians and other stakeholders concerned with emergency and healthcare planning in resource-constricted Sub-Saharan African countries.

## Introduction

Healthcare is an industry that directly affects people's lives at their most vulnerable moments. It is paramount to note that the perception of care is almost as important as the quality of care [1].

In modern-day medical care, delivering health care entails not only getting the patient well but also noting the overall experience of the patient while accessing health care. Patient satisfaction plays an ever-increasing role in the way hospitals are assessed. Although a lot of the public health systems are not overwhelmingly expensive and they are quite available, however, patients were still unsatisfied with the healthcare services [2]. This is a signal that patient satisfaction depends not only on the cost of accessing care but also on other factors bordering on quality of clinical services provided, availability of medications, behaviour of doctors and other paramedical staff, hospital infrastructure, physical comfort, emotional support and respect for patients' preferences [3]. Over the years, health caregivers noted that both clinical and non-clinical care influenced the overall consumers' satisfaction [4]. Patient satisfaction is an effective indicator to measure the success of health facilities and should be considered when designing the strategies for quality improvement of care.

Patient satisfaction though a subjective perception is considered to be the most important indicator of the quality of health care and has become a highly emphasised concept in the literature regarding emergency care [5,6]. It is also a marker of the quality of emergency care given in the Emergency Centre. However, the main factors affecting patient satisfaction are not yet fully understood [5]. This study therefore aimed at determining the satisfaction of patients and its associated factors with emergency care in Ekiti State University Teaching Hospital (EKSUTH), Ado Ekiti hoping that the result would guide the necessary changes needed for improvement.

## Methods

The study was conducted at the Emergency Centre of EKSUTH, Ado Ekiti in Southwest Nigeria. The centre is staffed with two consultants, ten medical officers who are experienced in Emergency Medicine. Twenty nursing officers who run an-eight-hour shift with a minimum of two nurses per shift and are assisted by 1–2 health assistants, a porter and an ambulance driver. All adult emergency cases are seen at the Emergency Centre, which functions 24 hours a day taking care of medical and surgical emergencies. There are 45 beds which are divided into sections: triage, red, yellow and green zones. The Medical officer on duty provides the initial treatment to all patients. After the initial assessment and stabilisation, patients can either receive emergency care and be discharged or further referred to the appropriate speciality for further management.

A validated questionnaire applied in a previous study in Nigeria by Ariba et al. was used [7]. The questionnaire contained eighteen items divided into four parts designed to evaluate the patients' perception of various aspects of care in the Emergency Centre. Family Medicine resident doctors and nursing students who were not primarily involved in the management of these patients were trained on how to conduct the interview and they administered the questionnaire at the point of discharge or transfer to the wards for further care.

The average number of total adult patients seen in the past year at the Emergency Centre was 2000, sampling 10% amounted to 200 patients for this study. Consequently, using a systematic sampling method, all adults 18 years and above that satisfied the inclusion criteria and willing to participate in the study were recruited except patients who were critically ill and those with altered mental states. The approval for this study was obtained from the Ethical review committee of the hospital with protocol number EKSUTH/A65/2018/006.

## Results

There were 199 respondents of which 52.3% were males and 47.7% were females. Majority of the patients (97.0%) were admitted for medical treatment while only 3.0% had surgical related illness. The length of stay at the Emergency Centre ranged between 1 and 6 days. Majority of the patients (60.3%) were admitted for only a day while 5.0% were admitted between 3 and 6 days.

Some of the patients were managed by the medical doctors at the Emergency Centre before discharge, while others were treated by the respective specialist doctors before discharge or transfer to the wards.

Table 1 shows the patients' assessment of the services at the Emergency Centre. Patient reception at the Emergency Centre was rated as very good by 51.3% of the respondents, (Table 1) while the speed of pain control was rated as excellent by only 9.0% of the respondents. About two-third (66.8%) rated the time to surgical intervention as more than good. Comparable rating was obtained by both doctors and nurses on the overall attitude across the 5 scoring domains with about half of the respondents (53.3% and 49.2%) scoring both (doctors and nurses) as very good.

**Table 1:** Patients' assessment at the Emergency Centre.

Variable	Excellent	Very good	Good	Fair	Poor
	n (%)	n (%)	n (%)	n (%)	n (%)
Reception at accident and emergency	33 (16.6)	102 (51.3)	58 (29.1)	6 (3.0)	0 (0.0)
Speed of pain control	18 (9.0)	115 (57.8)	58 (29.1)	7 (3.5)	1 (0.5)
Time to surgical or other intervention	19 (9.5)	114 (57.3)	58 (29.1)	8 (4.0)	0 (0.0)
Privacy	30 (15.1)	119 (59.8)	41 (20.6)	9 (4.5)	0 (0.0)
Doctors overall attitude	37 (18.6)	106 (53.3)	37 (18.6)	17 (8.5)	2 (1.0)
Nurses overall attitude	42 (21.1)	98 (49.2)	42 (21.1)	15 (7.5)	2 (1.0)
Clarity of instructions	37 (18.6)	96 (48.2)	56 (28.1)	9 (4.5)	1 (0.5)
Shown genuine concern by health workers	34 (17.1)	88 (44.2)	66 (33.2)	10 (5.0)	1 (0.5)
Adequacy of equipment	16 (8.0)	75 (37.7)	82 (41.2)	26 (13.1)	0 (0.0)
Courtesy by health workers	21 (10.6)	103 (51.8)	61 (30.7)	13 (6.5)	1 (0.5)
Rapidity of initial treatment	21 (10.6)	104 (52.3)	58 (29.1)	15 (7.5)	1 (0.5)

**Fig. 1:**
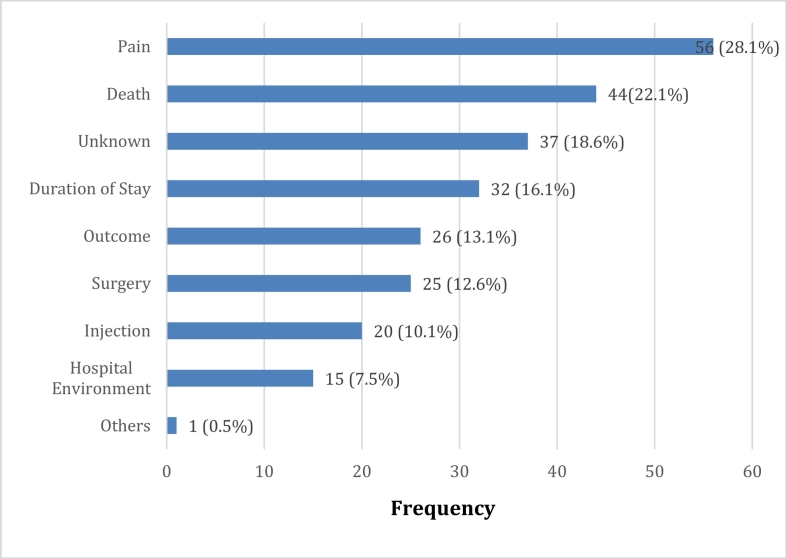
Fears of patients on arrival at the Emergency Centre.

Among the respondents, 4.0% reported been shouted at, while 4.5% were subjects of rude remarks and 8.0% reported of been spanked lightly or otherwise by the hospital workers. Although 98.0% of the participants had one on one discussion with a doctor, only 59.3% had their fears addressed. The important fears entertained by the patients included: pain (28.1%), death (22.1%) and long duration of stay in the hospital (16.1%) (Fig. 1).

Employment of more staff was the foremost (51.8%) on the areas suggested for improvement and it was closely followed by provision of more equipment (41.2%).

**Fig. 2:**
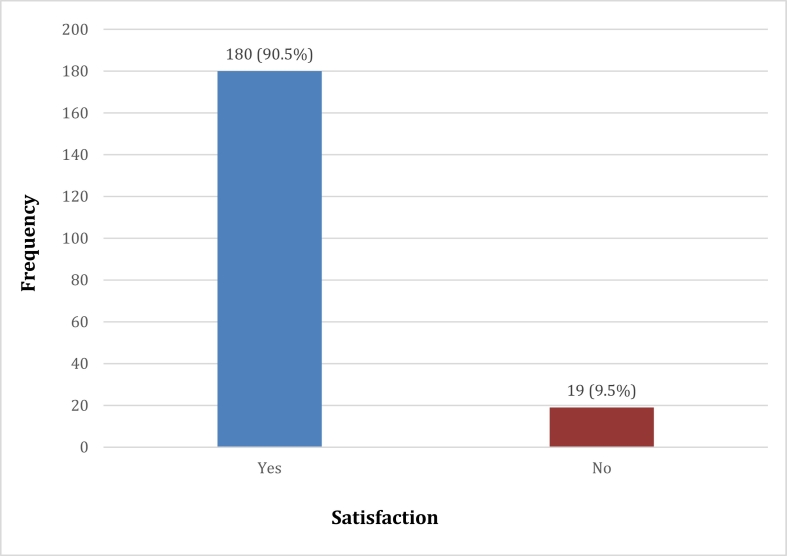
Overall patient satisfaction.

As shown in Fig. 2, 90.5% of patients were satisfied with the overall services and experiences at the Emergency Centre of the hospital. We did not find any significant association with patient satisfaction and the demographic characteristic of the participants, but it is worthy to note that a statistically significant association was found between patient satisfaction and experiencing rude remarks by hospital workers as well as having one on one discussion with doctors (P values 0.006 and 0.046 respectively). Patients who experienced rude remarks were less likely to be satisfied with the care received (52.6% vs 92.1%, P ≤ 0.0001).

Similarly, 91.3% of those who had one-on-one discussion with a doctor were satisfied with the care received as compared with only 50.0% of those who did not have such a discussion.

On multivariable analysis (using binary logistic regression), both experiences of being subject to rude remarks and having one-on-one discussion with a doctor remained significant predictors of patient satisfaction (P values 0.001 and 0.013). Patients who were subjected to rude remarks by hospital workers had lesser odds of being satisfied (OR: 0.094; 95% CI: 0.022–0.393) while having one on one discussion with a doctor, on the other hand, increased the odds of being satisfied (Table 2).

**Table 2:** Predictors of satisfaction with care at the Emergency Centre (multivariable analysis).

Variable	B	P value	OR (95% CI)
Being the subject of rude remarks by hospital workers	−2.365	0.001	0.094 (0.022–0.393)
Had one on one discussion with your doctor	2.588	0.013	13.308 (1.731–102.283)

B: coefficient of regression; OR: odds ratio; 95% CI: 95% confidence interval; predictive value: 90.5%.

## Discussion

It is a known fact that patients' assessment of the efficiency of services provided by a hospital is an important index of patient satisfaction [8]. Likewise, patient satisfaction has been reported to be a significant predictor of health-related behaviour such as utilisation of service and treatment compliance [9]. This study showed that the overall level of patient satisfaction was high 90.5% in our facility. The observed figure was higher than the studies done in some tertiary hospitals in Nigeria and Ethiopia that were between 78.5% and 86.7% [[10], 11., [12]]. Though this finding may not be surprising as studies had shown that patients generally tend toward expressing satisfaction with their care [7,12,13].

Also, the courteous cultural background of the society where our work was done could influence patient satisfaction. In this study, more than half of the participants indicated that the doctors' overall attitude was very good and about equal number reported the same for the nurses. In addition, 97.0% and 93.1% of the respondents in this study were satisfied with the reception at the Emergency Centre and courtesy of health workers respectively. This report agreed with the finding of Oyo-Ita et al. in Calabar, Nigeria where 81.4% of the respondents were satisfied with the attitude of the health workers [9]. Another study done in a Semi-urban community in South-western Nigeria by Abodunrin and his colleagues [14], revealed that 98.5% of the respondents were satisfied with the prompt attention received. However, in that study, about two-third (64.5%) of their participants rated the health workers' conduct as average in contrast to the finding from this study where the doctors and the nurses were respectively rated “good” by 18.6% and 21.1% respondents. Compared to the finding of Kampala et al. from Central Africa [15], Abodunrin et al. documented a lower rate of rude comments made to the patients in their study. This was shown by Kampala et al. to have a consequent negative effect on the health service utilisation. The differences recorded in these studies could be related to the different cultural background of the healthcare providers. We equally found a seemingly bad attitude among the healthcare workers where respondents reported light hitting by the caregivers. Though very few (8%) in this study attested to that as compared to 58.8% documented by Diya et al. in another study done in the emergency unit of a University Teaching Hospital, Southwest Nigeria [12].One would not be able to ascertain the reasons behind this; could it be that patients were not cooperating when carrying out some procedures such as giving a parenteral medication? Regardless of the reason, this attitude should be discouraged.

Good communication skill is vital to the quality of healthcare services given to patients as it increases their satisfaction. Majority of the respondents (98.0%) in this study had one-on-one discussion with their doctors, however, the significant fear of almost half of the respondents (40.7%) was not addressed. While fear is one of the major reasons why patients present in the hospital, the attending healthcare provider should recognise and address this symptom promptly. Effective healthcare providers- patients' communication is a vital element in patient-centred clinical care [12,16]. It is equally important to establish rapport and communicate effectively with the patient and various treatment options well explained. Providing training in this aspect of medicine (patient-centred clinical care) will be highly beneficial and effectively improve patient satisfaction as shown in previous studies [12,16,17].

Short waiting time has been identified to have a strong relationship with patient satisfaction. Previous studies showed that receiving prompt and rapid healthcare services was one of the most important determinants of overall patient satisfaction [12,17,18]. 92.0% of our respondents were satisfied with the rapidity of initial treatment given which is also in agreement with a local study by Diya et al. where the majority of the respondents were also satisfied with the initial treatment [12]. However, the short waiting time could be linked to the fact that the respondents in these studies were patients for emergency care.

Due to the small sample size and the fact that the majority of the respondents were medical patients, it is difficult to generalise the result from this study. Other services like proper hygiene on the wards and toilets, quality and quantity of food served were not included in the study.

## Conclusion

Even though the overall level of patient satisfaction in this study was 90.5%, however, areas suggested for improvement include the speed of pain control, inadequate staff and provision of more equipment and facilities. Communication is a major challenge throughout the world and if effectively done, it can improve patient satisfaction. Periodic patient satisfaction survey is crucial to the improvement of healthcare service delivery. The results from this study would be useful in implementing necessary changes needed to improve patient satisfaction in Emergency Centre.

## Dissemination of results

Results from this study were shared with staff members at the data collection site through an informal presentation.

## Authors’ contribution

Authors contributed as follows to the conception or design of the work; the acquisition, analysis, or interpretation of data, drafting or revising it critically for important intellectual content: DOO contributed 40%, DSA 35%, OJD 20% and SOA 5%. All authors approved the version to be published and agreed to be accountable to all aspects of the work.

## Declaration of competing interest

The authors declared no conflicts of interest.
